# Behavioral effects of ketamine and toxic interactions with psychostimulants

**DOI:** 10.1186/1471-2202-7-25

**Published:** 2006-03-16

**Authors:** Tamaki Hayase, Yoshiko Yamamoto, Keiichi Yamamoto

**Affiliations:** 1 Department of Legal Medicine, Kyoto University Graduate School of Medicine, Faculty of Medicine, Kyoto 606-8501, Japan; 2 Yamamoto Research Institute of Legal Medicine, Okazakitennou-cho, Sakyo-ku, Kyoto 606-8335, Japan

## Abstract

**Background:**

The anesthetic drug ketamine (KT) has been reported to be an abused drug and fatal cases have been observed in polydrug users. In the present study, considering the possibility of KT-enhanced toxic effects of other drugs, and KT-induced promotion of an overdose without making the subject aware of the danger due to the attenuation of several painful subjective symptoms, the intraperitoneal (i.p.) KT-induced alterations in behaviors and toxic interactions with popular co-abused drugs, the psychostimulants cocaine (COC) and methamphetamine (MA), were examined in ICR mice.

**Results:**

A single dose of KT caused hyperlocomotion in a low (30 mg/kg, i.p.) dose group, and hypolocomotion followed by hyperlocomotion in a high (100 mg/kg, i.p.) dose group. However, no behavioral alterations derived from enhanced stress-related depression or anxiety were observed in the forced swimming or the elevated plus-maze test. A single non-fatal dose of COC (30 mg/kg, i.p.) or MA (4 mg/kg, i.p.) caused hyperlocomotion, stress-related depression in swimming behaviors in the forced swimming test, and anxiety-related behavioral changes (preference for closed arms) in the elevated plus-maze test. For the COC (30 mg/kg) or MA (4 mg/kg) groups of mice simultaneously co-treated with KT, the psychostimulant-induced hyperlocomotion was suppressed by the high dose KT, and the psychostimulant-induced behavioral alterations in the above tests were reversed by both low and high doses of KT. For the toxic dose COC (70 mg/kg, i.p.)- or MA (15 mg/kg, i.p.)-only group, mortality and severe seizures were observed in some animals. In the toxic dose psychostimulant-KT groups, KT attenuated the severity of seizures dose-dependently. Nevertheless, the mortality rate was significantly increased by co-treatment with the high dose KT.

**Conclusion:**

Our results demonstrated that, in spite of the absence of stress-related depressive and anxiety-related behavioral alterations following a single dose of KT treatment, and in spite of the KT-induced anticonvulsant effects and attenuation of stress- and anxiety-related behaviors caused by COC or MA, the lethal effects of these psychostimulants were increased by KT.

## Background

The N-methyl-D-aspartate (NMDA) antagonist ketamine (KT) is an anesthetic drug used in veterinary practice [1-4]. However, recreational usage as a club drug has also been reported, and there are cases of fatal intoxication (e.g. cardiovascular and respiratory toxicity, etc.) particularly in polydrug users [5,6]. Recreationally used KT has been reported to cause euphoric hallucinations such as a feeling of dissociation of the mind from the body, and due to these psychotropic effects, the possibility of the disappearance of subjective symptoms accompanying toxicity has been suggested [7,8]. Furthermore, as this drug causes a loss of the ability to judge and induces amnesia, the possibility of its inappropriate usage (e.g. there has been an increased risk of use of KT as a date rape drug in criminally victimized individuals, etc.) has been reported [9,10].

Although animal models of complicated psychiatric symptoms have not been established, some favorable psychological effects (e.g. attenuations of behaviors related to pain such as anxiety-related behaviors and behavioral despair) have been reported [11,12]. On the other hand, an enhancement of the behavioral effects of other abused drugs (e.g. psychostimulants, etc.), which supports the increased risk of severe intoxication in human polydrug abusers, has been observed [13,14]. Therefore, in addition to a serious enhancement of the toxic effects of other drugs, it is possible that KT promotes cases of overdose by attenuating some painful subjective symptoms. In the present study, considering the importance of warning the danger associated with KT, the intraperitoneal (i.p.) KT-induced alterations in behaviors and toxic interactions with other popular drugs of abuse, the psychostimulants cocaine (COC) and methamphetamine (MA) [15,16], were examined in mice.

## Results

### Alterations in locomotor activity (Fig. 1)

**Figure 1:**
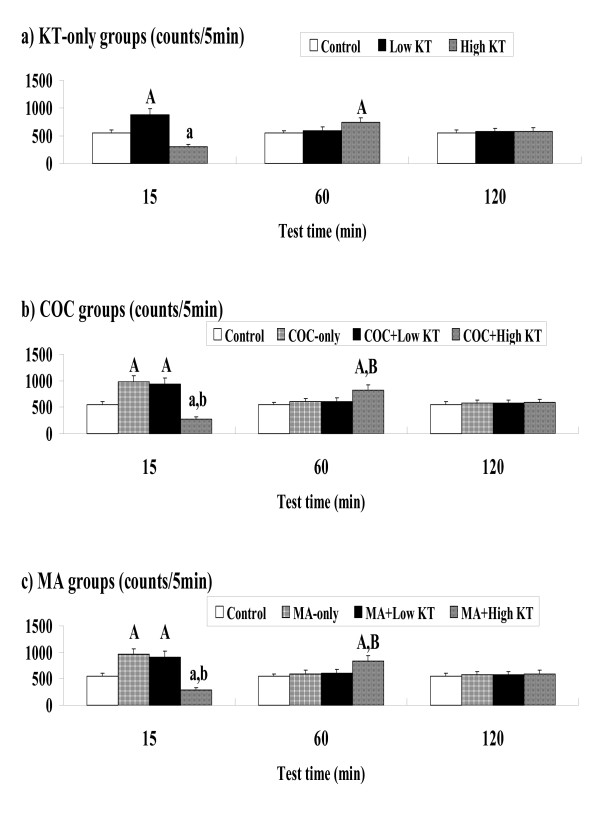
Locomotor activity measured at 15, 60 and 120 min time points. The data represent means ± SD (n = 8 for each group). A, a: significant increase (A) or attenuation (a) as compared to the control group. B, b: significant increase (B) or attenuation (b) as compared to the COC- or MA-only group.

For the KT-only groups (Fig. 1a), at 15 min time point, aggressive hyperlocomotion was observed and activity counts were increased as compared to the control group in the low KT (30 mg/kg)-only group, whereas hypolocomotion accompanied by a loss of the righting reflex was observed and activity counts were attenuated as compared to the control group in the high KT (100 mg/kg)-only group. At 60 min time point, recovery from hyperlocomotion was observed in the low KT-only group, whereas activity counts in the high KT-only group were significantly increased as compared to the control group until 120 min time point.

In the non-fatal dose COC (30 mg/kg)-only and MA (4 mg/kg)-only groups (Fig. 1b, 1c), only at 15 min time point, hyperlocomotion was observed and activity counts were increased as compared to the control group.

For the COC and MA groups of mice co-treated with KT (Fig. 1b, 1c), activity counts at 15 min time point were attenuated to levels significantly smaller than both the control and the psychostimulant-only groups, by co-treatment with the high dose (100 mg/kg) KT. Furthermore, in the psychostimulant plus high KT groups, activity counts were increased at 60 min time point as compared to both the control and the psychostimulant-only groups. On the other hand, in the psychostimulant plus low KT (30 mg/kg) groups (Fig. 1b, 1c), at 15 min time point, activity counts were increased to levels as large as the psychostimulant-only groups, and no reversing effects against the psychostimulant-induced hyperlocomotion were caused by KT.

The ANOVA analyses revealed significant main effects of KT treatment, psychostimulant treatment, and test time [see Additional file 1]. Furthermore, significant interactions between treatments and test times were also observed, which indicated some time-dependent and KT dose-dependent changes shown in Fig. 1.

### Alterations in stress-related depressive behaviors in the forced swimming test (Fig. 2)

**Figure 2:**
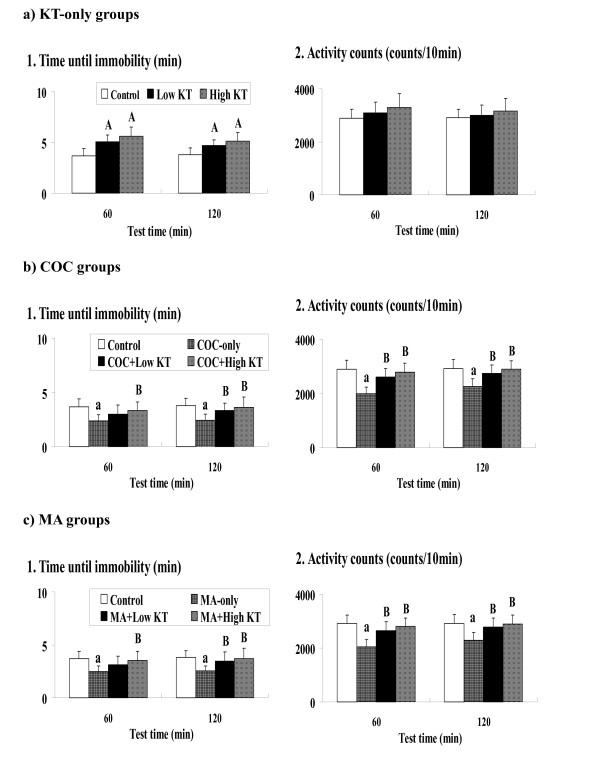
Alterations in stress-related depressive behaviors in the forced swimming test. The values for each parameter at 60 and 120 min time points are shown. The data represent means ± SD (n = 8 for each group). The symbols for differences are the same as the other figures.

For the KT-only groups (Fig. 2a), at any time points, no significant alterations derived from stress-related depression in swimming behaviors (attenuated time until immobility and attenuated activity counts) were observed in each parameter value, and the time until immobility was increased significantly as compared to the control group.

In the non-fatal dose COC (30 mg/kg)-only and MA (4 mg/kg)-only groups (Fig. 2b, 2c), stress-related depression in swimming behaviors was observed, and both time until immobility and activity counts were significantly attenuated.

For the COC and MA groups of mice co-treated with KT (Fig. 2b, 2c), recoveries from the alterations in each parameter value observed in the psychostimulant-only groups were provided by KT, and there were no significant differences as compared to the control group.

The ANOVA analyses revealed significant main effects of KT and psychostimulant treatments [see Additional file 1]. Although significant interactions between KT and psychostimulant treatments were not observed, recoveries shown in Fig. 2 were provided by KT in the COC and MA groups.

### Alterations in anxiety-related behaviors in the elevated plus-maze test (Fig. 3)

**Figure 3:**
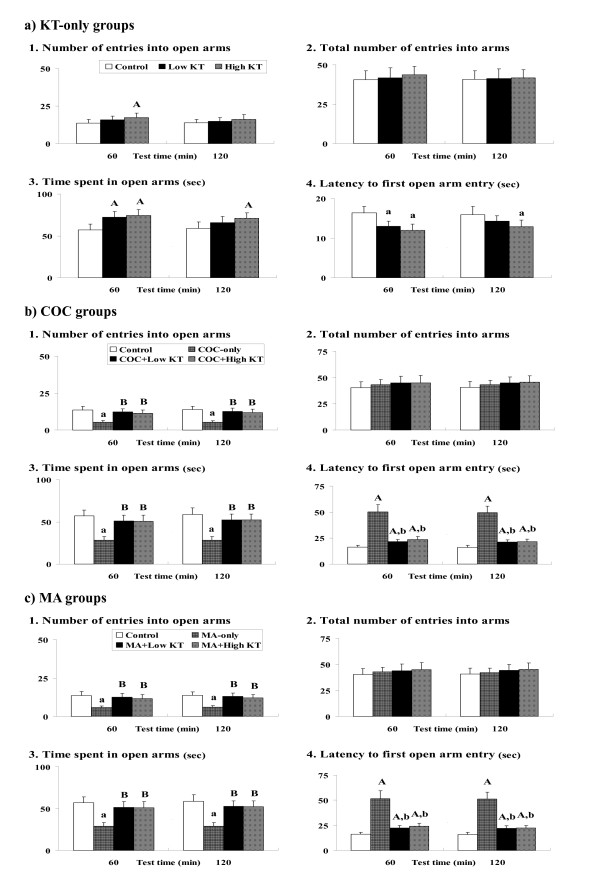
Alterations in anxiety-related behaviors in the elevated plus-maze test. The values for each parameter at 60 and 120 min time points are shown. The data represent means ± SD (n = 8 for each group). The symbols for differences are the same as the other figures.

For the KT-only groups (Fig. 3a), at any time points, no significant behavioral alterations derived from anxiety-related preference for closed arms (attenuated number of entries into and time spent in open arms, and increased latency to first open arm entry) were observed in each parameter value. On the other hand, the number of entries into open arms and the time spent in open arms tended to be increased, and the latency to first open arm entry tended to be attenuated (Fig. 3a-1, 3a-3, 3a-4).

In the non-fatal dose COC (30 mg/kg)-only and MA (4 mg/kg)-only groups (Fig. 3b, 3c), anxiety-related behaviors were observed: the number of entries into and time spent in open arms were attenuated, and the latency to first open arm entry was increased.

For the COC and MA groups of mice co-treated with KT (Fig. 3b, 3c), recoveries from the alterations in each parameter value observed in the psychostimulant-only groups were provided by KT, and there were no significant differences, except for increased latency to first open arm entry, as compared to the control group.

For the locomotor activity on the plus-maze, no quantitative alterations were observed, and there were no significant differences in total number of entries into arms between the groups (Fig. 3a-2, 3b-2, 3c-2).

The ANOVA analyses revealed significant main effects of KT and psychostimulant treatments, and significant interactions between KT and psychostimulant treatments, for the number of entries into and time spent in open arms, and the latency to first open arm entry [see Additional file 1]. However, for the total number of entries into arms, no significant effects or interactions were observed.

### Alterations in toxic effects of high dose COC and MA (Fig. 4)

**Figure 4:**
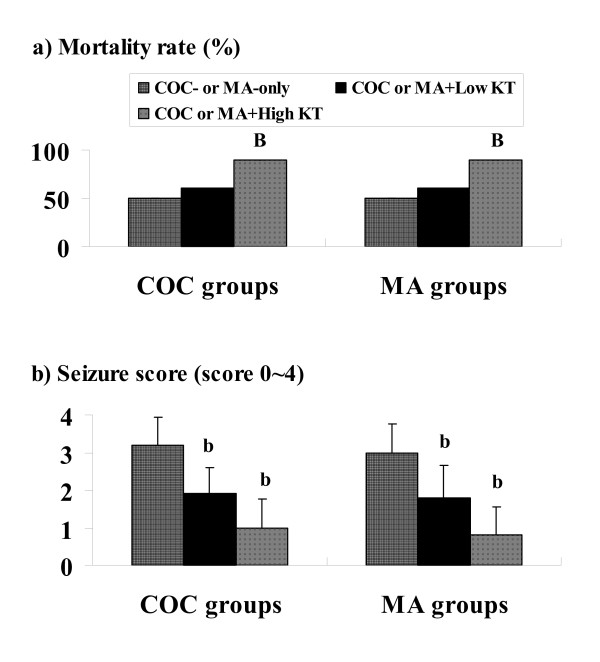
Alterations in mortality rate (%) and seizure scores (means ± SD) in the toxic COC and MA groups (n = 10 for each group). The symbols for differences are the same as the other figures. In the KT-only groups, neither mortality nor seizures were observed (not presented in the figure).

For the toxic dose COC (70 mg/kg) and MA (15 mg/kg) groups, mortality and severe seizures were observed in some animals even in the psychostimulant-only groups (Fig. 4), whereas neither mortality nor seizures were observed in the KT-only groups.

In the groups of mice co-treated with KT, the mortality rate tended to be increased and was significantly increased by co-treatment with the high dose KT (Fig. 4a), although the seizure scores were attenuated significantly by both low and high doses of KT, in a dose-dependent manner (Fig. 4b).

Using a 3 (low dose KT, high dose KT versus vehicle) × 2 (toxic dose of psychostimulant COC versus MA) factorial design, the ANOVA analyses revealed significant main effects of KT treatment on seizure scores [see Additional file 1].

## Discussion

The anesthetic effects of KT have been reported to be closely correlated with its antagonistic effects on NMDA receptors [17,18], but persistent modifications of other neurons including dopamine (DA), norepinephrine (NE), and serotonin (5-HT) neurons have also been reported to accompany the behavioral effects of KT [13,14,19]. These KT-induced modifications of the neurons in the group of mice treated with a single KT are different depending on the dosage [19]: hyperlocomotion observed in both low and high dose KT groups (Fig. 1a) has been reported to be correlated with a DA agonist action induced by KT, but with high KT doses which caused hypolocomotion during the early period (Fig. 1a), the activation of NE and 5-HT neurons has also been suggested. In the group of mice treated with a single COC or MA, the psychostimulant which exerted KT-like DA agonistic actions [13,14], hyperlocomotion was also observed (Fig. 1b, 1c). Furthermore, in the COC- and MA-only groups, unlike in the KT-only groups, both stress-related depressive behaviors in the forced swimming test (attenuated time until immobility and attenuated activity counts in the swimming behaviors) and anxiety-related behaviors in the elevated plus-maze test (preference for closed arms, that is, attenuated number of entries into and time spent in open arms, and increased latency to first open arm entry) were observed (Fig. 2b, 2c, 3b, 3c). In the KT-only groups, there were no such stress-related depressive and anxiety-related behavioral alterations (Fig. 2a, 3a). On the contrary, the parameter values in the forced swimming test tended to be increased by KT at levels above those in the control group, which indicated an attenuation of the stress-related depressive behavioral alterations below the control level (Fig. 2a). Furthermore, the KT-induced alterations in parameter values in the elevated plus-maze test (tendency of increased number of entries into and time spent in open arms, and attenuated latency to first open arm entry as compared to the control group) indicated a preference for open arms, that is, an attenuation of anxiety-related behavioral alterations below the control level (Fig. 3a). The mechanisms responsible for these behavioral alterations have not been elucidated. For the psychostimulant-only groups, the contribution of the neurons which have been reported to be correlated with stress-related depressive and anxiety-related behaviors (e.g. DA and benzodiazepine neurons) [20-23] can be predicted. For the KT-only groups, it is possible that the behavioral alterations caused by KT were correlated with the antagonistic effects of KT on NMDA receptors, considering the fact that NMDA antagonist actions have been demonstrated at both low and high doses of KT [24,25]. The contributions of other modified neurons such as DA, NE, and 5-HT neurons [19] can also be suspected.

In the non-fatal dose psychostimulant groups of mice co-treated with KT, the stress-related depressive and anxiety-related behavioral alterations caused by the psychostimulants were antagonized consistently by both high and low doses of KT (Fig. 2b, 2c, 3b, 3c). The alterations in locomotor activity were also antagonized by the high dose KT (Fig. 1b, 1c). In our preliminary experiments, the stress- and anxiety-related behavioral alterations were antagonized less effectively by MK-801, a more selective and a high affinity antagonist for NMDA receptors, which exerted stronger antagonistic effects on the locomotor activity than KT [26-28]. Considering these preliminary results, contributions of other neurons such as DA, NE, and 5-HT neurons, for which some modifications have been reported in both psychostimulant and KT treatments [19,21,23,29,30], could be suspected for the antagonistic effects of KT. However, further experimental studies are required for elucidating the mechanisms related to these neurons for the different behavioral alterations in each test. In humans, although some unfavorable subjective effects such as dysphoria have been observed depending on the dose [31], a characteristic euphoric mind-altering effect, which may persist subsequent to the appearance of the "peaceful out-of-body" feeling (a feeling of dissociation of the mind from the body), has been reported for the recreational use of KT [5,32]. This sense of euphoric transcendence in humans, which has not been reported for other amnestic drugs such as thiopental and has been suspected to be the predominant reason for the dependence on and abuse of KT as a club drug [32], cannot be identified with the attenuation of stress- and anxiety-related behavioral alterations in mice shown in the present experiments. However, the attenuation of painful subjective effects including stress and anxiety has been reported to increase a preference for drugs in humans [33], and may promote a dangerous polydrug abuse such as a psychostimulant plus KT abuse [5,6]. For the KT-only treatment, although no significant behavioral abnormalities or fatal toxicities were observed at or after 120 min time point in our animal model, it is possible that the absence of stress- and anxiety-related behavioral alterations promotes further use of this drug in humans. Furthermore, for the use of KT, a significantly greater incidence of abnormalities of mental status has been reported than for the use of the other amnestic drugs [34,35]. In rats, a delayed occurrence of some abnormal behaviors similar to those observed in the model of schizophrenia has been reported for the KT treatment groups in several behavioral tests [36].

In the groups of mice treated with KT plus a toxic dose COC or MA, the drug frequently co-abused with KT in overdose cases [15,16], KT significantly enhanced the fatality caused by COC or MA in spite of its anticonvulsant effects. The anticonvulsant effects of KT against COC- or MA-induced seizures seem to be correlated with NMDA receptors, based on its antagonistic effects against NMDA-induced convulsions and the reported contribution of NMDA receptors to psychostimulant-induced seizures [37-39]. Furthermore, considering the fact that the anticonvulsant effects of KT were as strong as MK-801 in our preliminary experiments, it is possible that receptors other than NMDA receptors contributed to these effects. For example, a contribution of GABAA receptors is also predictable based on the antagonistic effects of KT against the GABAA antagonist bicuculline-induced seizures and the reported contribution of GABAA receptors to psychostimulant-induced seizures [40-42]. With respect to the increase in mortality rate despite the anticonvulsant effects of KT, the underlying mechanisms have not been elucidated. However, it is possible that KT directly enhanced the psychostimulant-induced cardiovascular and respiratory toxicity. Considering that the behavioral effects mediated by DA and NE neurons were enhanced by combining COC with KT [13,14], the toxic peripheral effects (e.g. cardiovascular effects, etc.) mediated by these neurons may also be enhanced by the psychostimulant-KT combination. Therefore, it is possible that KT, like ethanol, a drug which increases the lethal effects of COC and for which behavioral effects similar to KT have been reported [43-45], enhances the toxic effects of psychostimulants without causing severe convulsions or painful subjective symptoms such as anxiety.

## Conclusion

Our results support the contention that the recreational use of KT is dangerous because, in addition to its toxic effects with or without combined drugs of abuse, the accompanying attenuation of some painful subjective symptoms such as anxiety and stress-related depression may accelerate the use of the drugs and may promote an overdose without making the subject aware of the danger.

In our experiments, in addition to the absence of any stress-related depressive and anxiety-related behavioral alterations following a single dose KT in the forced swimming and elevated plus maze tests, a KT-induced attenuation of stress- and anxiety-related behaviors caused by COC or MA was demonstrated. The scores of the seizures caused by toxic doses of COC or MA were also attenuated by KT. Nevertheless, the lethal effects caused by toxic doses of these psychostimulants were increased by KT.

## Methods

### Animals and drug treatments

Male ICR mice (60–90 days old) (Shizuoka Laboratory Animal Center, Hamamatsu, Japan) were housed in a forced-air facility which was maintained at 23°C and 50% relative humidity, with a 12 h/12 h light/dark cycle [46,47]. The mice were kept separately in single transparent cages measuring 23.5 × 16.5 × 12 cm, and were allowed water and rodent chow *ad libitum *[46,47]. The experiments described in this report were conducted in accordance with the "Guidelines for Animal Experiments" of our institution (1988), which are based on the National Institutes of Health Guide for Care and Use of Laboratory Animals, and any pain experienced by the mice was minimized.

The dose of ketamine hydrochloride (KT) (Sankyo, Co., Ltd., Tokyo, Japan) was selected based on preliminary experiments and previous studies examining its behavioral effects [11-13,19]. Considering the dose-dependent effects on various neurons [13,14,19], the experiments were performed for both low (30 mg/kg) and high (100 mg/kg) doses (low and high KT groups).

For experiments examining the interactions between KT and psychostimulants, the doses of cocaine hydrochloride (COC) (Takeda Chemical Industries, Ltd., Osaka, Japan) and methamphetamine hydrochloride (MA) (Dainippon Pharmaceutical, Co., Ltd., Osaka, Japan) simultaneously administered with KT [13,14] were selected based on preliminary experiments and previous studies on their behavioral effects [47-52]. After preliminary experiments, the COC and MA doses causing effects with similar peak severity in the behavior and toxicity tests were selected. For the evaluation of the behavioral effects, a single dose of 4 mg/kg MA or 30 mg/kg COC was administered with KT. For the evaluation of the toxic effects, a single dose of 15 mg/kg MA or 70 mg/kg COC was administered with KT.

For each treatment, i.p. injections were performed. KT, COC, and MA were administered dissolved in saline to a volume of 5 ml/kg. In the KT-only groups, the same volumes of saline vehicle were injected instead of COC or MA. In the COC-only and MA-only groups, the same volumes of saline vehicle were injected instead of KT. In the control group without any drug treatment (control group), the same volumes of saline vehicle were injected instead of both psychostimulants and KT. The drug administration and each experimental session were performed between 17 and 21 hrs light cycle.

### Evaluation of alterations in locomotor activity

Based on previous studies [13,19] and preliminary experiments, alterations (5 min test periods) in locomotor activity caused by the low dose (30 mg/kg) and high dose (100 mg/kg) KT were observed, and activity counts were evaluated at 15, 60, and 120 min after the drug treatment using the activity-measuring and recording system Supermex-CompACT AMS instrument (Muromachi Kikai Co. Ltd., Tokyo, Japan) [47,52].

### Evaluation of alterations in behavioral despair (stress-related depressive behaviors) in the forced swimming test

Based on previous studies [47,52-55], alterations in behavioral despair in the forced swimming test, which had been proved to indicate stress-related behavioral alterations, were examined using a glass cylinder apparatus 33 cm in height and 18 cm in diameter containing 14 cm of water at 21–23°C. The time until immobility (the time after when only modest swimming behaviors necessary to avoid drowning were observed) and the activity counts for 10 min yielded by swimming behaviors were evaluated at 60 and 120 min after drug treatment. During the earlier time point (within 60 min after drug treatment), behavioral effects of the drugs (e.g. a loss of the righting reflex, etc.) interfered with the swimming behaviors, and exposed the mice to the danger of drowning. The activity was counted using the Supermex-CompACT AMS instrument by placing the sensor of the instrument over the cylinder at a distance of 20 cm from the water.

### Evaluation of alterations in anxiety-related behaviors in the elevated plus-maze test

Based on previous studies [48,52,56], alterations in anxiety-related behaviors were examined by the elevated plus-maze test using an apparatus that consisted of two opposite open arms 50 × 10 cm (length and width) and two closed arms 50 × 10 × 30 cm (length, width, and height). As parameters for the test (5 min test periods), the number of entries into open arms, the total number of entries into arms, the time spent in open arms (sec), and the latency to first open arm entry (sec) were evaluated at 60 and 120 min after drug treatment. During the earlier time point (within 60 min after drug treatment), due to the notable alterations in locomotor activity, correct values for anxiety-related behavioral parameters on the plus-maze could not be obtained. The parameters were evaluated after placing each mouse diagonally in the center of the maze, facing both the open and closed arms.

### Evaluation of effects on the toxicity of psychostimulants

Using a toxic dose of COC (70 mg/kg) or MA (15 mg/kg), the effects of KT on mortality and seizures were examined. The mortality rate (%) and the seizure score (score 0: absence of seizures; score 1: short-lasting mild seizures; score 2: short-lasting seizures with a loss of the righting reflex; score 3: convulsive seizures accompanied by severe clonus or rearing; and score 4: severe convulsive seizures continuous and violent enough to cause fatal respiratory disorders), the score for the most severe episode of seizures in each mouse, were evaluated based on previous studies [52,57].

### Statistical analysis

The data obtained for each treatment at each time point were subjected to three-way or two-way analysis of variance (ANOVA) for the factors KT treatment × psychostimulant treatment × test time, or the factors KT treatment × psychostimulant treatment, using a 3 (low dose KT, high dose KT versus vehicle) × 3 (psychostimulant COC, MA versus vehicle) × 3 (15 min, 60 min versus 120 min) factorial design for the evaluation of locomotor activity, a 3 (low dose KT, high dose KT versus vehicle) × 3 (psychostimulant COC, MA versus vehicle) × 2 (60 min versus 120 min) factorial design for the parameters in the forced swimming and elevated plus-maze tests, and a 3 (low dose KT, high dose KT versus vehicle) × 2 (psychostimulant COC versus MA) factorial design for the seizure score [22]. For the parameter values from the forced swimming and elevated plus-maze tests, the results of ANOVA analyses are summarized in the table [see Additional file 1]. For pairwise comparisons, post-hoc Bonferroni tests were performed [58]. For comparisons of the mortality rate, Fisher's exact test was used [52,55]. All of the comparisons were performed using statistical software packages ('Excel Statistics' from Social Survey Research Information Co. Ltd. Inc., Tokyo, Japan). Unless otherwise noted, P values less than 0.05 were considered to be statistically significant.

## Authors' contributions

TH designed and performed the behavioral experiments. YY and KY advised and improved the methods based on their previous or preliminary experiments, and also participated partly in the experiments. All authors read and approved the final manuscript.

## Supplementary Material

Additional file 1 Summary of statistical analyses. F values with the degrees of freedom are shown. Significant effects and interactions are noted: * P < 0.05, ** P < 0.01, *** P < 0.001. Click here for file

## References

[B1] Dundee JW, Knox JW, Black GW, Moore J, Pandit SK, Bovill J, Clarke RS, Love SH, Elliott J, Coppel DL (1970). Ketamine as an induction agent in anaesthetics. Lancet.

[B2] Wright M (1982). Pharmacologic effects of ketamine and its use in veterinary medicine. J Am Vet Med Assoc.

[B3] Hahn N, Eisen RJ, Eisen L, Lane RS (2005). Ketamine-medetomidine anesthesia with atipamezole reversal: practical anesthesia for rodents under field conditions. Lab Anim.

[B4] Nonaka A, Suzuki S, Masamune T, Imamura M, Abe F (2005). Anesthetic management by total intravenous anesthesia with propofol, pentazocine and ketamine. Masui.

[B5] Fitzpatrick M (2003). Special K. Lancet.

[B6] Gable RS (2004). Acute toxic effects of club drugs. J Psychoactive Drugs.

[B7] Pandit SK, Kothary SP, Kumar SM (1980). Low dose intravenous infusion technique with ketamine. Amnesic, analgesic and sedative effects in human volunteers. Anaesthesia.

[B8] Hansen G, Jensen SB, Chandresh L, Hilden T (1988). The psychotropic effect of ketamine. J Psychoactive Drugs.

[B9] Jansen KL (1993). Non-medical use of ketamine. BMJ.

[B10] Smith KM (1999). Drugs used in acquaintance rape. J Am Pharm Assoc.

[B11] Babar E, Ozgunen T, Melik E, Polat S, Akman H (2001). Effects of ketamine on different types of anxiety/fear and related memory in rats with lesions of the median raphe nucleus. Eur J Pharmacol.

[B12] Yilmaz A, Schulz D, Aksoy A, Canbeyli R (2002). Prolonged effect of an anesthetic dose of ketamine on behavioral despair. Pharmacol Biochem Behav.

[B13] Vanderwende C, Spoerlein MT, Lapollo J (1982). Cocaine potentiates ketamine-induced loss of the righting reflex and sleeping time in mice. Role of catecholamines. J Pharmacol Exp Ther.

[B14] Uchihashi Y, Kuribara H, Tadokoro S (1992). Assessment of the ambulation-increasing effect of ketamine by coadministration with central-acting drugs in mice. Jpn J Pharmacol.

[B15] Gill JR, Stajic M (2000). Ketamine in non-hospital and hospital deaths in New York City. J Forensic Sci.

[B16] Riley SC, James C, Gregory D, Dingle H, Cadger M (2001). Patterns of recreational drug use at dance events in Edinburgh, Scotland. Addiction.

[B17] Thomson AM, West DC, Lodge D (1985). An N-methylaspartate receptor-mediated synapse in rat cerebral cortex: a site of action of ketamine?. Nature.

[B18] Irifune M, Shimizu T, Nomoto M, Fukuda T (1992). Ketamine-induced anesthesia involves the N-methyl-D-aspartate receptor-channel complex in mice. Brain Res.

[B19] Irifune M, Shimizu T, Nomoto M (1991). Ketamine-induced hyperlocomotion associated with alteration of presynaptic components of dopamine neurons in the nucleus accumbens of mice. Pharmacol Biochem Behav.

[B20] Van der Meersch-Mougeot V, da Rocha M, Monier C, Diquet B, Puech AJ, Thiebot MH (1993). Benzodiazepines reverse the anti-immobility effect of antidepressants in the forced swimming test in mice. Neuropharmacology.

[B21] Cancela LM, Basso AM, Martijena ID, Capriles NR, Molina VA (2001). A dopaminergic mechanism is involved in the 'anxiogenic-like' response induced by chronic amphetamine treatment: a behavioral and neurochemical study. Brain Res.

[B22] Paine TA, Jackman SL, Olmstead MC (2002). Cocaine-induced anxiety: alleviation by diazepam, but not buspirone, dimenhydrinate or diphenhydramine. Behav Pharmacol.

[B23] Hayase T, Yamamoto Y, Yamamoto K, Muso E, Shiota K, Hayashi T (2003). Similar effects of cocaine and immobilization stress on the levels of heat-shock proteins and stress-activated protein kinases in the rat hippocampus, and on swimming behaviors: the contribution of dopamine and benzodiazepine receptors. Behav Pharmacol.

[B24] Bell RF (1999). Low-dose subcutaneous ketamine infusion and morphine tolerance. Pain.

[B25] Duncan GE, Miyamoto S, Leipzig JN, Lieberman JA (1999). Comparison of brain metabolic activity patterns induced by ketamine, MK-801 and amphetamine in rats: support for NMDA receptor involvement in responses to subanesthetic dose of ketamine. Brain Res.

[B26] Yamakura T, Mori H, Masaki H, Shimoji K, Mishina M (1993). Different sensitivities of NMDA receptor channel subtypes to non-competitive antagonists. Neuroreport.

[B27] Berman FW, Murray TF (1996). Characterization of [3H]MK-801 binding to N-methyl-D-aspartate receptors in cultured rat cerebellar granule neurons and involvement in glutamate-mediated toxicity. J Biochem Toxicol.

[B28] Uzbay IT, Wallis CJ, Lal H, Forster MJ (2000). Effects of NMDA receptor blockers on cocaine-stimulated locomotor activity in mice. Behav Brain Res.

[B29] Xu F, Gainetdinov RR, Wetsel WC, Jones SR, Bohn LM, Miller GW, Wang YM, Caron MG (2000). Mice lacking the norepinephrine transporter are supersensitive to psychostimulants. Nat Neurosci.

[B30] Bubar MJ, McMahon LR, De Deurwaerdere P, Spampinato U, Cunningham KA (2003). Selective serotonin reuptake inhibitors enhance cocaine-induced locomotor activity and dopamine release in the nucleus accumbens. Neuropharmacology.

[B31] Smith DJ, Bouchal RL, deSanctis CA, Monroe PJ, Amedro JB, Perrotti JM, Crisp T (1987). Properties of the interaction between ketamine and opiate binding sites in vivo and in vitro. Neuropharmacology.

[B32] Pal HR, Berry N, Kumar R, Ray R (2002). Ketamine dependence. Anaesth Intensive Care.

[B33] Chutuape MA, de Wit H (1994). Relationship between subjective effects and drug preferences: ethanol and diazepam. Drug Alcohol Depend.

[B34] Garfield JM, Garfield FB, Stone JG, Hopkins D, Johns LA (1972). A comparison of psychologic responses to ketamine and thiopental – nitrous oxide – halothane anesthesia. Anesthesiology.

[B35] Moretti RJ, Hassan SZ, Goodman LI, Meltzer HY (1984). Comparison of ketamine and thiopental in healthy volunteers: effects on mental status, mood, and personality. Anesth Analg.

[B36] Becker A, Peters B, Schroeder H, Mann T, Huether G, Grecksch G (2003). Ketamine-induced changes in rat behaviour: A possible animal model of schizophrenia. Prog Neuropsychopharmacol Biol Psychiatry.

[B37] Derlet RW, Albertson TE, Rice P (1990). Antagonism of cocaine, amphetamine, and methamphetamine toxicity. Pharmacol Biochem Behav.

[B38] Sofia RD, Gordon R, Gels M, Diamantis W (1994). Comparative effects of felbamate and other compounds on N-methyl-D-aspartic acid-induced convulsions and lethality in mice. Pharmacol Res.

[B39] Witkin JM, Gasior M, Heifets B, Tortella FC (1999). Anticonvulsant efficacy of N-methyl-D-aspartate antagonists against convulsions induced by cocaine. J Pharmacol Exp Ther.

[B40] Bachus SE, Gale K (1986). Muscimol microinfused into the nigrotegmental target area blocks selected components of behavior elicited by amphetamine or cocaine. Naunyn Schmiedebergs Arch Pharmacol.

[B41] Velíšková J, Velíšek L, Mareš P, Rokyta R (1990). Ketamine suppresses both bicuculline- and picrotoxin-induced generalized tonic-clonic seizures during ontogenesis. Pharmacol Biochem Behav.

[B42] Ye JH, Liu PL, Wu WH, McArdle JJ (1997). Cocaine depresses GABAA current of hippocampal neurons. Brain Res.

[B43] Grant KA, Knisely JS, Tabakoff B, Barrett JE, Balster RL (1991). Ethanol-like discriminative stimulus effects of non-competitive n-methyl-d-aspartate antagonists. Behav Pharmacol.

[B44] Sanger DJ (1993). Substitution by NMDA antagonists and other drugs in rats trained to discriminate ethanol. Behav Pharmacol.

[B45] Krystal JH, Petrakis IL, Webb E, Cooney NL, Karper LP, Namanworth S, Stetson P, Trevisan LA, Charney DS (1998). Dose-related ethanol-like effects of the NMDA antagonist, ketamine, in recently detoxified alcoholics. Arch Gen Psychiatry.

[B46] Boyer CS, Petersen DR (1992). Enzymatic basis for the transesterification of cocaine in the presence of ethanol: evidence for the participation of microsomal carboxylesterases. J Pharmacol Exp Ther.

[B47] Hayase T, Yamamoto Y, Yamamoto K (2000). Stress-related behavioral alterations accompanying cocaine toxicity: the effects of mixed opioid drugs. Nihon Arukoru Yakubutsu Igakkai Zasshi.

[B48] Hayase T, Yamamoto Y, Yamamoto K (2005). Persistent anxiogenic effects of a single or repeated doses of cocaine and methamphetamine: interactions with endogenous cannabinoid receptor ligands. Behav Pharmacol.

[B49] Rogerio R, Takahashi RN (1992). Anxiogenic properties of cocaine in the rat evaluated with the elevated plus-maze. Pharmacol Biochem Behav.

[B50] Hayase T, Yamamoto Y, Yamamoto K, Abiru H, Nishitani Y, Fukui Y (1999). Effects of ethanol and/or cardiovascular drugs on cocaine- and methamphetamine-induced fatal toxicities in mice. Nihon Arukoru Yakubutsu Igakkai Zasshi.

[B51] Szumlinski KK, Haskew RE, Balogun MY, Maisonneuve IM, Glick SD (2001). Iboga compounds reverse the behavioural disinhibiting and corticosterone effects of acute methamphetamine: Implications for their antiaddictive properties. Pharmacol Biochem Behav.

[B52] Hayase T, Yamamoto Y, Yamamoto K (2003). Brain excitatory amino acid transporters (EAATs) and treatment of methamphetamine toxicity. Nihon Arukoru Yakubutsu Igakkai Zasshi.

[B53] Porsolt RD, Le Pichon M, Jalfre M (1977). Depression: a new animal model sensitive to antidepressant treatments. Nature.

[B54] Porsolt RD, Anton G, Blavet N, Jalfre M (1978). Behavioural despair in rats: a new model sensitive to antidepressant treatments. Eur J Pharmacol.

[B55] Hayase T, Yamamoto Y, Yamamoto K (2002). Toxic cocaine- and convulsant-induced modification of forced swimming behaviors and their interaction with ethanol: comparison with immobilization stress. BMC Pharmacol.

[B56] Prior H, Schwegler H, Marashi V, Sachser N (2004). Exploration, emotionality, and hippocampal mossy fibers in nonaggressive AB/Gat and congenic highly aggressive mice. Hippocampus.

[B57] Przewlocka B, Lason W, Machelska H, Przewlocki R (1994). The effects of cocaine-induced seizures on the proenkephalin mRNA level in the mouse hippocampus: a possible involvement of the nitric oxide pathway. Neurosci Lett.

[B58] Alves SH, Pinheiro G, Motta V, Landeira-Fernandez J, Cruz AP (2004). Anxiogenic effects in the rat elevated plus-maze of 5-HT(2C) agonists into ventral but not dorsal hippocampus. Behav Pharmacol.

