# Supplementary material for: Behavioral effects of ketamine and toxic interactions with psychostimulants
Source: BMC Neurosci. 2006 Mar 16;7:25. doi: 10.1186/1471-2202-7-25 (PMC1473192; doi:10.1186/1471-2202-7-25)
Supplement: Additional file 1 — Summary of statistical analyses. F values with the degrees of freedom are shown. Significant effects and interactions are noted: * P < 0.05, ** P < 0.01, *** P < 0.001. [file 1471-2202-7-25-S1.doc]

a) Locomotor activity, parameters in the forced swimming test, and seizure score

|  | Locomotor activity | Parameters in the forced swimming test | | Seizure score |
| --- | --- | --- | --- | --- |
|  |  | Time until immobility | Activity counts |  |
| KT treatment | F(2, 189)=55.77*** | F(2, 126)=30.51*** | F(2, 126)=30.64*** | F(2, 54)=36.83*** |
| Psychostimulant treatment | F(2, 189)=17.74*** | F(2, 126)=63.07*** | F(2, 126)=29.64*** | F(1, 54)=0.63 |
| Test time | F(2, 189)=31.94*** | F(1, 126)=0.17 | F(1, 126)=1.93 | ― |
| KT × psychostimulant treatment interaction | F(4, 189)=8.69*** | F(4, 126)=0.68 | F(4, 126)=1.96 | F(2, 54)=0.03 |
| KT treatment × test time interaction | F(4, 189)=209.63*** | F(2, 126)=0.04 | F(2, 126)=0.58 | ― |
| Psychostimulant treatment × test time interaction | F(4, 189)=5.51*** | F(2, 126)=1.31 | F(2, 126)=1.62 | ― |
| KT × psychostimulant treatment × test time interaction | F(8, 189)=8.16*** | F(4, 126)=0.43 | F(4, 126)=0.01 | ― |

b) Parameters in the elevated plus-maze test

|  | Parameters in the elevated plus-maze test | | | |
| --- | --- | --- | --- | --- |
|  | Number of entries into open arms | Total number of entries into arms | Time spent in open arms | Latency to first open arm entry |
| KT treatment | F(2, 126)=74.35*** | F(2, 126)=1.77 | F(2, 126)=130.63*** | F(2, 126)=374.70*** |
| Psychostimulant treatment | F(2, 126)=78.72*** | F(2, 126)=2.87 | F(2, 126)=171.87*** | F(2, 126)=299.34*** |
| Test time | F(1, 126)=0.005 | F(1, 126)=0.01 | F(1, 126)=0.04 | F(1, 126)=0.42 |
| KT × psychostimulant treatment interaction | F(4, 126)=7.01*** | F(4, 126)=0.04 | F(4, 126)=4.39** | F(4, 126)=67.23*** |
| KT treatment × test time interaction | F(2, 126)=0.06 | F(2, 126)=0.01 | F(2, 126)=0.22 | F(2, 126)=0.20 |
| Psychostimulant treatment × test time interaction | F(2, 126)=0.74 | F(2, 126)=0.08 | F(2, 126)=1.16 | F(2, 126)=0.52 |
| KT × psychostimulant treatment × test time interaction | F(4, 126)=0.17 | F(4, 126)=0.10 | F(4, 126)=0.69 | F(4, 126)=0.14 |
